# WearSense: Detecting Autism Stereotypic Behaviors through Smartwatches

**DOI:** 10.3390/healthcare5010011

**Published:** 2017-02-28

**Authors:** Amir Mohammad Amiri, Nicholas Peltier, Cody Goldberg, Yan Sun, Anoo Nathan, Shivayogi V. Hiremath, Kunal Mankodiya

**Affiliations:** 1 Department of Physical Therapy, College of Public Health, Temple University, Philadelphia, PA 19140, USA; shiv.hiremath@temple.edu; 2 Department of Electrical, Computer, and Biomedical Engineering, University of Rhode Island, Kingston, RI 02881, USA; npeltier@my.uri.edu (N.P.); joncgoldberg@gmail.com (C.G.); yansun@ele.uri.edu (Y.S.); kunalm@uri.edu (K.M.); 3 Smart Monitor Co., San Jose, CA 95119, USA; anoo@smart-monitor.com

**Keywords:** autism, m-health, smartwatch, ASD, activity recognition

## Abstract

Autism is a complex developmental disorder that affects approximately 1 in 68 children (according to the recent survey conducted by the Centers for Disease Control and Prevention—CDC) in the U.S., and has become the fastest growing category of special education. Each student with autism comes with her or his own unique needs and an array of behaviors and habits that can be severe and which interfere with everyday tasks. Autism is associated with intellectual disability, impairments in social skills, and physical health issues such as sleep and abdominal disturbances. We have designed an Internet-of-Things (IoT) framework named WearSense that leverages the sensing capabilities of modern smartwatches to detect stereotypic behaviors in children with autism. In this work, we present a study that used the inbuilt accelerometer of a smartwatch to detect three behaviors, including hand flapping, painting, and sibbing that are commonly observed in children with autism. In this feasibility study, we recruited 14 subjects to record the accelerometer data from the smartwatch worn on the wrist. The processing part extracts 34 different features in each dimension of the three-axis accelerometer, resulting in 102 features. Using and comparing various classification techniques revealed that an ensemble of 40 decision trees has the best accuracy of around 94.6%. This accuracy shows the quality of the data collected from the smartwatch and feature extraction methods used in this study. The recognition of these behaviors by using a smartwatch would be helpful in monitoring individuals with autistic behaviors, since the smartwatch can send the data to the cloud for comprehensive analysis and also to help parents, caregivers, and clinicians make informed decisions.

Autism spectrum disorder (ASD) refers to a group of complex neurodevelopmental disorders that can be characterized by repetitive and characteristic patterns of behavior, and difficulties with social communication and interaction. The symptoms typically appear in early childhood and affect the individual’s ability to communicate and interact with others. According to the latest report from the Centers for Disease Control and Prevention in 2014, 1 in 68 children in the United States is diagnosed with ASD, which is almost twice as high as 1 in 125 for 2004 [1]. Caring for a child with autism is expensive; the scaled cost of caring for a child with autism for a lifetime is estimated to be as significant as $2.4 million [2], and the Autism Society has estimated that around $90 billion is invested for costs of autism in the United States annually [3]. This shows the need for more extensive research in the field, and the research needs to be conducted during early stages to improve the quality of life for individuals with autism.

The symptoms of Autism can be categorized into three groups: social development, communication, and repetitive behaviors; i.e., flapping or stimming. These stereotypic behaviors happen when a child is trying to regulate the sensory input from their surrounding environment. For a long time, it was assumed that these three characteristic symptoms have a common cause at the genetic, cognitive, and neural levels. However, ongoing research has indicated that autism is a convoluted disorder with specific causes that often co-occur [4].

The goal of our work is to establish and test an Internet of things (IoT) framework named WearSense that could leverage the sensing capabilities of modern smartwatches in detecting and monitoring such behaviors to facilitate clinical assessment. WearSense consists of a smartwatch, a smartphone with an app to collect the accelerometer sensor data, and machine learning algorithms to detect and classify the repetitive behaviors (see Figure 1).

We have conducted a feasibility study on 12 healthy and 2 pathological participants who were asked to perform three tasks involving repetitive behaviors observed in autism. The objectives of the study were:

- To evaluate the performance of WearSense in recording the stereotypic movements;
- To develop a set of suitable classifiers that could robustly classify the stereotypic movements;
- To compare and correlate the classifiers for the data collected from 14 participants who present their individual variations.

We believe that in the future WearSense will help monitor stereotypic behaviors in naturalistic settings when individuals with autism are performing their daily activities at homes or schools. Successful monitoring of repetitive behaviors can significantly contribute to critical life-quality-improvement tasks, such as evaluation of responsiveness to therapies and prediction of disruption autism actions.

## 1. Related Work

Many studies have been performed to develop and evaluate tools for the recognition of physical and emotional activity for individuals with autism.

In particular, N. Mohammadian Rad [5] presented a wireless inertial sensing technology that offers an infrastructure for real-time Stereotypical Motor Movements (SMM) detection. The automation would provide support for tuned intervention and possibly early alert on the onset of meltdown events. The authors developed automatic SMM detection systems based on a deep learning architecture. Authors proposed to employ the deep learning paradigm in order to learn the discriminating features from multi-sensor accelerometer signals.

Recently, another study by A. Coronato [6,7] reported a method and an infrastructure for the detection of the stereotyped motion disorders of patients with ASD. The method adopted artificial intelligence techniques such as Artificial Neural Network (ANN) for the identification of stereotyped motion disorders and the Situation-Awareness paradigm for the reduction of misclassification and the extraction information from accelerometer signals. Quantitatively, the off-line classifier has shown an accuracy of over 99%; whereas the on-line classifier has an accuracy of 92%.

In another study by M. S. Goodwin et al. [8,9,10], authors used three-axis accelerometer data obtained through wearable wireless sensors. The data was collected from six individuals on the autism spectrum. The authors compared pattern recognition results for different classifiers such as Support Vector Machine and Decision Tree, in combination with different feature sets based on time–frequency characteristics of accelerometer data. they achieved accuracy rates over 90% for SMM such as hand flapping and body rocking across subjects.

Inspired by the work of [11], researchers focus on automatically detecting SMMs in real-time considering two different approaches. The first approach uses the Microsoft sensor Kinect and gesture recognition algorithms, and the second approach uses a trademark device of Texas Instruments with built-in accelerometers and statistical methods to recognize stereotyped movements. The two proposed systems were tested in children with ASD, and the results were compared.

Our review on the state of the art shows extensive usage of various feature extraction approaches and machine learning algorithms from the accelerometer signals. In the current study, according to our consult with the specialist in autism school, the use of a sensor and wristband may increase the risk of an autism attack; we therefore used a smartwatch, which was more convenient for the children with autism to wear. Additionally, a set of features in time and frequency domains is reported which was obtained by using a combination of signal processing tools such as Wigner-Ville Bispectrum and Wigner-Ville Trispectrum. In this paper, we propose a method for automatically classifying accelerometer signals based on the three most common SMMs using an ensemble of decision trees to identify autism stereotypic behaviors.

## 2. WearSense Architecture

The Autism data were collected in two phases. (i) We recruited 12 healthy subjects aged between 23–33 and 165 samples collected data. We asked them to simulate some of these stereotypic behaviors, commonly observed in autism; (ii) We also recruited two subjects (ages 15 and 16) diagnosed with autism. These two subjects (one male, one female) were students of the J. Arthur Trudeau Memorial Center Pathways program, RI. Pathways is a classroom located in Cranston High School West, focused on autistic children at the high school age level. The smart watch recorded sensor data from the subjects as they went about their normal day. Data was recorded from these two subjects over the course of many months, with average trial sessions typically lasting from one to two hours. Trial Notes were simultaneously taken with observations and time stamps of what was happening with the client wearing the smart watch. These notes gave the researchers an idea as to which portions of data would prove useful to our research.

**Figure 1:**
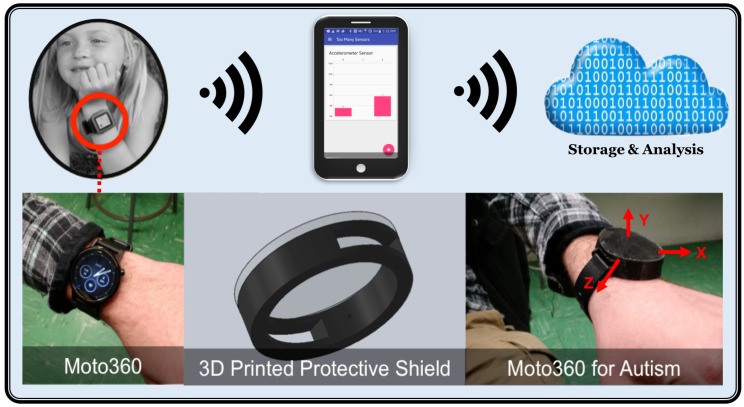
WearSense system architecture for monitoring autism behavior activity.

The tasks that the subjects were invited to do included three different types for 20 s. The first task was “flapping” their hands in front of their face. The second task was “painting” a common task of writing or drawing on a piece of paper which represents one of the daily life activity that is performed by children or adults with autism. The third task was “sibbing”, which means hitting themselves on the top of their head. The WearSense architecture includes three aspects:

**Smartwatch:** During the whole procedure of recording the data, the participants wore a Moto360 SmartWatch protected by a 3D-printed shield. The Moto360 carries several sensors for recording the data; in this work, an accelerometer sensor with a sampling rate of 50 Hz was used. The smartwatch sent the data to a smartphone via Bluetooth and saved the data on the smartphone (see Figure 1).

**Smartphone App:** An Android app was prepared with both a smartphone and smartwatch component, written in Kotlin 1.0.0. Google Play services provide communication between both devices. The phone queries the watch for a list of all the possible sensors, and the watch responds, populating the sensor list on the phone. Users can select any number of these sensors to record from. Once started, the UI displays all data in real-time, and the data is also saved in a .CSV format for later use.

**Figure 2:**
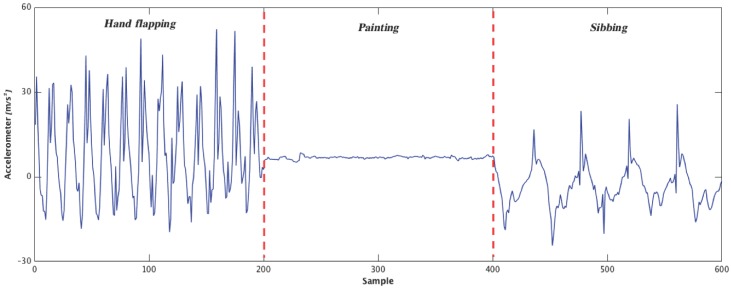
Illustration of Z-axis variation of the accelerometer for three activities.

**Cloud Computing:** The accelerometer is an electromechanical device that measures the acceleration forces, which could be static or dynamic. Measuring the static acceleration reveals the angle that the device is placed on the earth, and then we can find out how and in which direction the device is moving by measuring the amount of dynamic acceleration. The accelerometer signals are usually in three dimensions—*X*, *Y*, and *Z*. An example of the accelerometer signal in three different activities is shown in Figure 2. The accelerometer signals from the smartphone are analyzed in the cloud through the use of classification algorithms such as an ensemble of decisions trees that are explained in the next section.

## 3. Methods

The data was cleaned and analyzed by extracting features that highlight important properties of movement. A set of 102 features were extracted from 165 samples of 2 s. Regarding feature extraction, several metrics were taken into account, including Maximum and Minimum value amplitudes, Peak to Peak, Variance, Entropy, Fast Fourier Transform, Discrete Cosine Transform, Z-transform, Bispectrum, Wigner-Ville Bispectrum and Trispectrum, which have proved to be very useful for improving classification performance. For this reason, let us spend a few words on the most essential techniques.

### 3.1. Bispectrum

The power spectrum is based on the second-order statistics of the time series, and the third-order spectrum, called Bispectrum [12]. In particular, Bispectrum is an example of a higher-order spectrum (HOS), which is defined as the Fourier transform of third-order cumulant sequence [13]. If the signal is a stationary random process with real values, then it can be defined as follows [14]:

(1) $$B\left(\omega \right)=X\left(\omega_{1}\right).X\left(\omega_{2}\right).X^{*}\left(\omega_{1},\omega_{2}\right)$$

As Bispectrum analysis is not easy to calculate, and it shows signal into $\omega_{1}$ and $\omega_{2}$ frequency domains, this slice of spectrum obtained from a Bispectrum is used to assess whether the analysis of data exhibits nonlinear or Gaussian distribution in the signal.

### 3.2. Wigner-Ville Bispectrum and Trispectrum

The Wigner-Ville distribution (WVD) is one class of bilinear distributions which transfers signal into time–frequency domains capabilities [15].

The WVD has multiple extensions, one including the Wigner high-order spectrum (WHOS). The WHOS keeps the advantages the WVD intact, but also the benefits of the HOS. These spectra have been used in both non-stationary and non-Gaussian realms, signifying its use for analyzing accelerometer signals. WVD combined with WHOS is able to extract time and frequency information simultaneously [16].

The higher-order spectra of WVD of order *k* of a complex deterministic signal x(t) can be defined as follows [17]:

(2) $$\begin{array}{cc} W_{kx}(t, & f_{1},f_{2},\cdots ,f_{k})=\\ & \int_{\tau_{1}}\cdots \int_{\tau_{k}}x^{*}\left(t-\frac{1}{k+1}\sum_{m=1}^{k}\tau_{m}\right)\\ & \cdot \prod_{i=1}^{k}x\left(t+\frac{k}{k+1}\tau_{i}-\frac{1}{k+1}\sum_{j=1,j\neq i}^{k}\tau_{i}\right)\\ & \cdot \prod_{i=1}^{k}exp\left(-2j\pi f_{i}\tau_{i}\right)\;d\tau_{1}d\tau_{2}\cdots d\tau_{k}\;\;, \end{array}$$

where $W_{kx}\left(t,f_{1},f_{2},\cdots ,f_{k}\right)$ is a *k*-dimensional Fourier transform of a *k*-dimensional local moment function as $R_{kt}\left(\tau_{1},\cdots ,\tau_{k}\right)$ described in [16]. According to the Equation (2), special cases of WHOS include Wigner Bispectrum (WB) for k=2 is obtained:

(3) $$\begin{array}{cc} W_{2x}(t, & f_{1},f_{2})=\int_{\tau_{1}}\int_{\tau_{2}}\\ & x^{*}\left(t-\frac{1}{3}\tau_{1}-\frac{1}{3}\tau_{2}\right)\cdot x\left(t+\frac{2}{3}\tau_{1}-\frac{1}{3}\tau_{2}\right)\\ & \cdot x\left(t+\frac{2}{3}\tau_{2}-\frac{1}{3}\tau_{1}\right)\cdot exp\left(-j2\pi f_{1}\tau_{1}\right)\\ & \cdot exp\left(-j2\pi f_{2}\tau_{2}\right)\;d\tau_{1}d\tau_{2}, \end{array}$$

and we propose the following definition for the Wigner Trispectrum (WT) for k=3:

(4) $$\begin{array}{cc} W_{3x}(t, & f_{1},f_{2},f_{3})=\int_{\tau_{1}}\int_{\tau_{2}}\int_{\tau_{3}}\\ & x^{*}\left(t-\frac{1}{4}\tau_{1}-\frac{1}{4}\tau_{2}-\frac{1}{4}\tau_{3}\right)\\ & \cdot x\left(t+\frac{3}{4}\tau_{1}-\frac{1}{4}\tau_{2}-\frac{1}{4}\tau_{3}\right)\\ & \cdot x\left(t+\frac{3}{4}\tau_{2}-\frac{1}{4}\tau_{1}-\frac{1}{4}\tau_{3}\right)\\ & \cdot x\left(t+\frac{3}{4}\tau_{3}-\frac{1}{4}\tau_{1}-\frac{1}{4}\tau_{2}\right)\cdot exp\left(-j2\pi f_{1}\tau_{1}\right)\\ & \cdot exp\left(-j2\pi f_{2}\tau_{2}\right)\cdot exp\left(-j2\pi f_{3}\tau_{3}\right)\;d\tau_{1}d\tau_{2}d\tau_{3}. \end{array}$$

In the real-valued case, the accelerometer signals can be recovered from this projection. The WB and WT are analyzed for use as inputs in a classifier.

## 4. Classification

To classify autistic behavior activities, an ensemble of decision trees—performed by Bagging method—is used. Decision tree (DT) is a nonparametric classification method, and its structure is like a tree, wherein it starts from the topmost by a root node and splits into two branches. DT decides whether to assign a class label to the node or to recursively split the node into two internal nodes which denote a test on an attribute. The outgoing branches of a node represent an outcome of the test [18,19].

**Figure 3:**
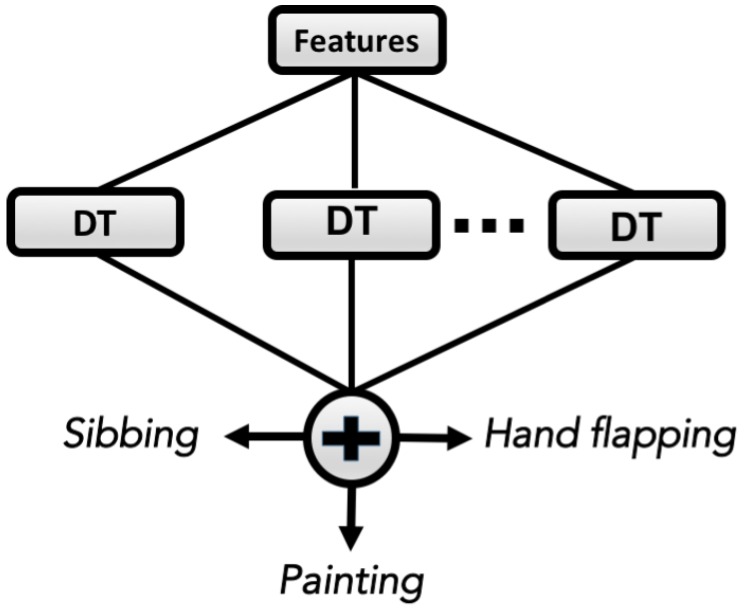
Schematic of the ensemble of decision trees (DTs) made by 40 DTs.

The Bagging methods are proposed for the construction of a set of individual decision trees. Bagging is one of the most efficient ensemble learning methods, used in this work to increase the prediction accuracy of DTs. Leo Breiman, 1996 presented this approach, and Bagging creates an ensemble of classifiers by sampling with replacement from training data set to bring a new training set called “bags” [20] (see Figure 3).

## 5. Experiments and Results

This section reports the experimental results, which have been tested to analyze the proposed method. Our first step was to extract the features of accelerometer signals, as the performance of the classifiers mostly depends on selecting proper features. The feature set is 102 which was obtained by using various signal processing tools in time–frequency domains as mentioned in the Methods section. The ensemble of decision trees is proposed as a classifier, where the final ensemble consists of 40 trees (Figure 3).

*K*-fold cross validation (K=10) has been used for training and validation (testing). In this method, a feature set is split into *K* parts of approximately equal sizes with the K−1 part used to develop a model through training, and the remaining *K*^th^ part is used for testing. This process is repeated *K* times [21].

Experiments have been performed on a balanced set of 165 samples gained by windowing signals into a 2-s window (meaning that the number of samples was the same for each activity). The overall accuracy was calculated by averaging the results obtained over 10 times run of DT ensembles. Classification results of the ensemble of DT are shown in Table 1 in the form of a confusion matrix, together with the percentage of each classification accuracy.

It can be seen that all samples of hand flapping and painting were classified correctly, and out of 50 sibbing tasks, 45 (90%) were correctly classified as sibbing, and 5 (10%) were misclassified as hand flapping. The classification results verify the validity and performance of the proposed algorithm to recognizing autistic behavior activity with 94.6% accuracy.

**Figure 4:**
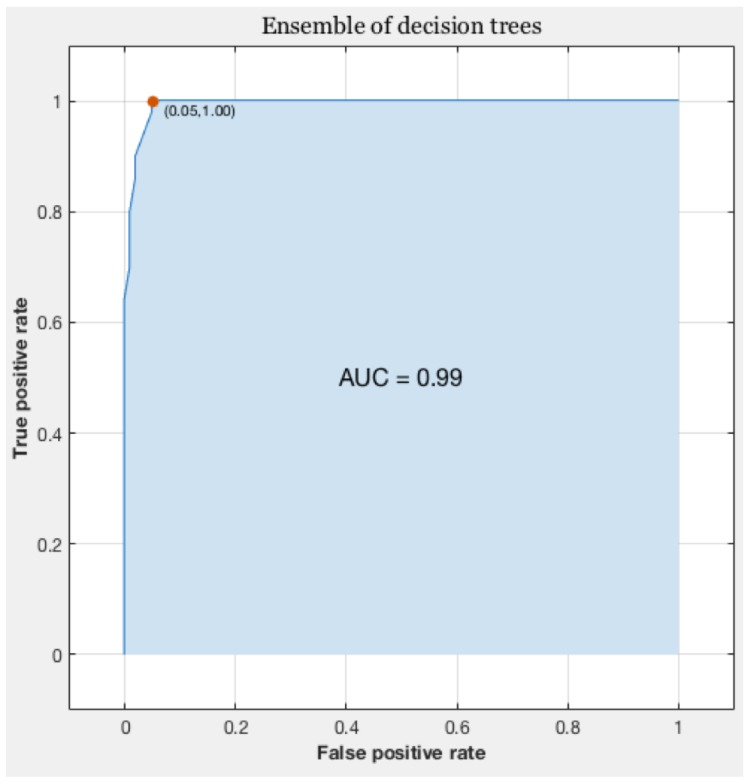
Receiver Operating Characteristic (ROC) curve of autism behavior activity recognition.

The Receiver Operating Characteristic (ROC) curve is for a binary classification problem, the true positive rate is reported as a function of the false positive rate for different cut-off points. A classifier with perfect performance has an area under the curve (AUC) equal to 1 (see Figure 4).

Table 2 shows the results in comparison with other classifiers regarding accuracy, the area under ROC, and training time. *K*-fold cross-validation has been used for all classifiers.

## 6. Conclusions

The aim of this study was to establish a smartwatch-based system to recognize and monitor the autism behavior activity which may be harmful to the person. The novelty of the methods and algorithms used in this study shows that it is possible to record the motion data by a smartwatch which can easily communicate with a smartphone and send data to the cloud for future processing. The full range of features was extracted from the data, and an ensemble of DTs was used that led to an accuracy of 96.7% in the recognition of the autistic behaviors. In particular, combining classifiers using majority votes turned out to be a superior classifier for classifying three autism actions. The findings in this paper can be exceptionally helpful for monitoring and serving children who have autism. 

## Figures and Tables

**Table 1:** Classification result of autism behavior activity.

Classes	Flapping	Painting	Sibbing	Accuracy
Flapping	51 (93%)	0 (0%)	4 (8%)	93%
Painting	0 (0%)	55 (100%)	0 (0%)	100%
Sibbing	5 (9%)	0 (0%)	50 (90%)	91%
Average/Overall		165		94.6%

**Table 2:** Comparison of accuracy, area under the curve (AUC), and training time for different classifiers.

Model	Accuracy	AUC	Training Time
Complex Tree	87%	0.84	0.9 s
Simple Tree	83.0%	0.83	0.8 s
Linear SVM	33.3%	0.5	2.7 s
Gaussian SVM	54.2%	0.78	1.4 s
Ensemble (Boosted Trees)	68.8%	0.76	6.6 s
Ensemble (Bagged Trees)	94.6%	0.99	7.3 s
